# Project G-SPACE: protocol for exploring the influence of green space on sleep and mental health among children

**DOI:** 10.1186/s12887-024-05247-3

**Published:** 2024-11-29

**Authors:** Diana S. Grigsby-Toussaint, Jong Cheol Shin, Aliana Rodriguez Acevedo, William Kemball-Cook, Diane Story, Abby Katz, Ugoji Nwanaji-Enwerem, Gabrielle Evans, Azia Johnson, Brooke Ury, Yaideliz M. Romero-Ramos, Jue Yang, David M. Barker, John E. McGeary, Shira I. Dunsiger

**Affiliations:** 1 Department of Behavioral and Social Sciences, Department of Epidemiology, Center for Health Promotion and Health Equity, School of Public Health, Brown University, Providence, RI, USA; 2 Community and Population Health, Lehigh University, Bethlehem, PA, United States; 3 Psychiatry and Human Behavior, Warren Alpert School of Medicine, Brown University, Providence, RI, United States; 4 Providence VA Medical Center, Providence, RI, United States; 5 Research Center, Hasbro Children’s Hospital, Providence, RI, United States

**Keywords:** Green space, Sleep, Mental health, Children, Actigraphy, Physical activity, Well-being, Epigenetics, Elementary school

## Abstract

**Background:**

The prevention of pediatric mental health disorders is a growing health priority in the United States. While exposure to green space, such as outdoor vegetation, has been linked with improved mental health outcomes in children, little is known about the impact of green space on children’s sleep. Sleep has many benefits, but the factors affecting both sleep and mental health as they relate to green space exposure are not well understood in children. This study aims to investigate how green space can affect sleep in children and contribute to the promotion of mental health and wellbeing.

**Methods:**

Project Green Space, Sleep, and Mental Health (G-SPACE) aims to recruit 250 elementary school-children from first, second, and third grade in Rhode Island to examine the influence of green space exposure on sleep, physical activity, and mental health over a five-year period. Objective measures of sleep, physical activity, and daily activity space will be assessed using an actigraph and a GPS (Global Positioning System) unit. Subjective measures of sleep duration, sleep quality, and mental health will be assessed using daily sleep diaries from parents, in addition to a range of survey items, including PROMIS^®^ (Patient Reported Outcome Measurement Information System) pediatric scales, and the Children’s Sleep Habits questionnaire, among others. Green space exposure will be based on measures of green space from the normalized difference vegetation index (NDVI) aligned with the daily activity trajectory of children. Additionally, saliva and DNA samples will be collected to examine epigenetic mechanisms linking green space to sleep and mental health. A subset of participants (*n* = 50) will be followed longitudinally to evaluate the long-term impact of green space on sleep and mental health among children. Multi-level models will be used to assess the association between green space exposure, sleep behaviors, and mental health.

**Discussion:**

Project G-SPACE will evaluate whether green space utilization influences sleep and mental health in early elementary school children, and the possible mechanistic pathways through which these associations emerge.

## Background

### Pediatric mental health

Globally, more than 10% children and youth aged 5 to 24 years and 17% of those aged 2 to 8 years currently live with a diagnosable mental disorder [1, 2]. Pediatric mental health conditions were associated with $31 billion in United States healthcare spending and 46.6% of all pediatric medical spending in 2021 [3]. The lifetime prevalence of anxiety in American children aged 3 to 17 is 9.4%, while the lifetime prevalence of depression is 4.4% [4]. Additionally, 8.9% of children in the same group experience behavioral or conduct problems [4]. The development of mental illness symptoms is multifactorial, however, sleep is increasingly recognized as a significant determinant [5–16]. High-quality sleep has been shown to improve attention, behavior, emotional regulation, and general mental and physical health among children [17–21]. Conversely, children with mental health problems such as attention-deficit/hyperactivity disorder (ADHD), anxiety, depression, and behavioral disorders (e.g., oppositional defiant disorder) are more likely to have nocturnal awakenings, long sleep latency, excessive daytime sleepiness, and bedtime struggles [22–25]. In addition, 37.4% of children aged 6 to 12 sleep less than the recommended amount for their age group, thereby exacerbating the burden of pediatric mental illness [26]. The link between sleep and mental health has been recognized for some time, yet there is limited understanding of the factors that influence sleep and mental health among pediatric populations [27]. Potential determinants of sleep in children include electronic device use, socioeconomic status, daily mood, stress, and traumatic events [28–31]. Furthermore, the neighborhood environment has been demonstrated to impact child sleep patterns through mechanisms such as safety concerns and limited opportunities for physical activity (PA) [32].

### Green space, physical activity, sleep, and mental health

#### Green space and mental health

Exposure to green space, broadly defined as various forms of outdoor vegetation, has been found to be restorative and associated with enhanced health and well-being in children [33–39]. Specifically, green space exposure has been shown to improve coping with life events and cognitive functioning, while also decreasing the prevalence of negative mental health symptoms and physical illness (e.g., obesity) [40–45]. Green space is also linked to improved mood and has been shown to reduce stress levels [37–39], serving as a buffer for life stress [45] in children.

#### Sleep & physical activity

There is mixed evidence regarding the relationship between physical activity (PA) and sleep in children. One study found a bi-directional relationship between high PA and poor sleep quality [46]. Other evidence suggests that PA increases sleep efficiency [47]. High-intensity PA has been shown to elevate the proportion of slow-wave sleep, improve sleep efficiency, and shorten sleep onset latency [48, 49]. In general, a systematic review of the literature suggests that exercise enhances sleep quality or duration across various age groups [50].

#### Sleep, physical & mental health

Poor sleep quality, sleep disorders, and sleep deprivation can have negative consequences on physical and mental health for people of all ages [13, 15]. Sleep problems have been associated with various mental health issues, including depression [11], emotional dysfunction [21, 23], and psychiatric disorders [24]. Insomnia has been linked to depression and anxiety [9, 12], increased risk behaviors [21], and has been found to predict paranoia [6, 17], as well as negatively influence the course of severe mental illnesses such as bipolar disorder and schizophrenia [10]. Sleep alterations have been observed across multiple severe mental disorders [12]. A systematic review focused on adolescents revealed that inadequate sleep increased the risk of obesity, was linked to poor general health, was associated with poor academic performance, and led to a higher tendency toward risky behaviors [21]. Furthermore, inadequate sleep in children and adolescents leads to daytime sleepiness, which can impair their behavior, mood, and performance [20, 22], while also contributing to poor self-regulation [19].

**Fig. 1:**
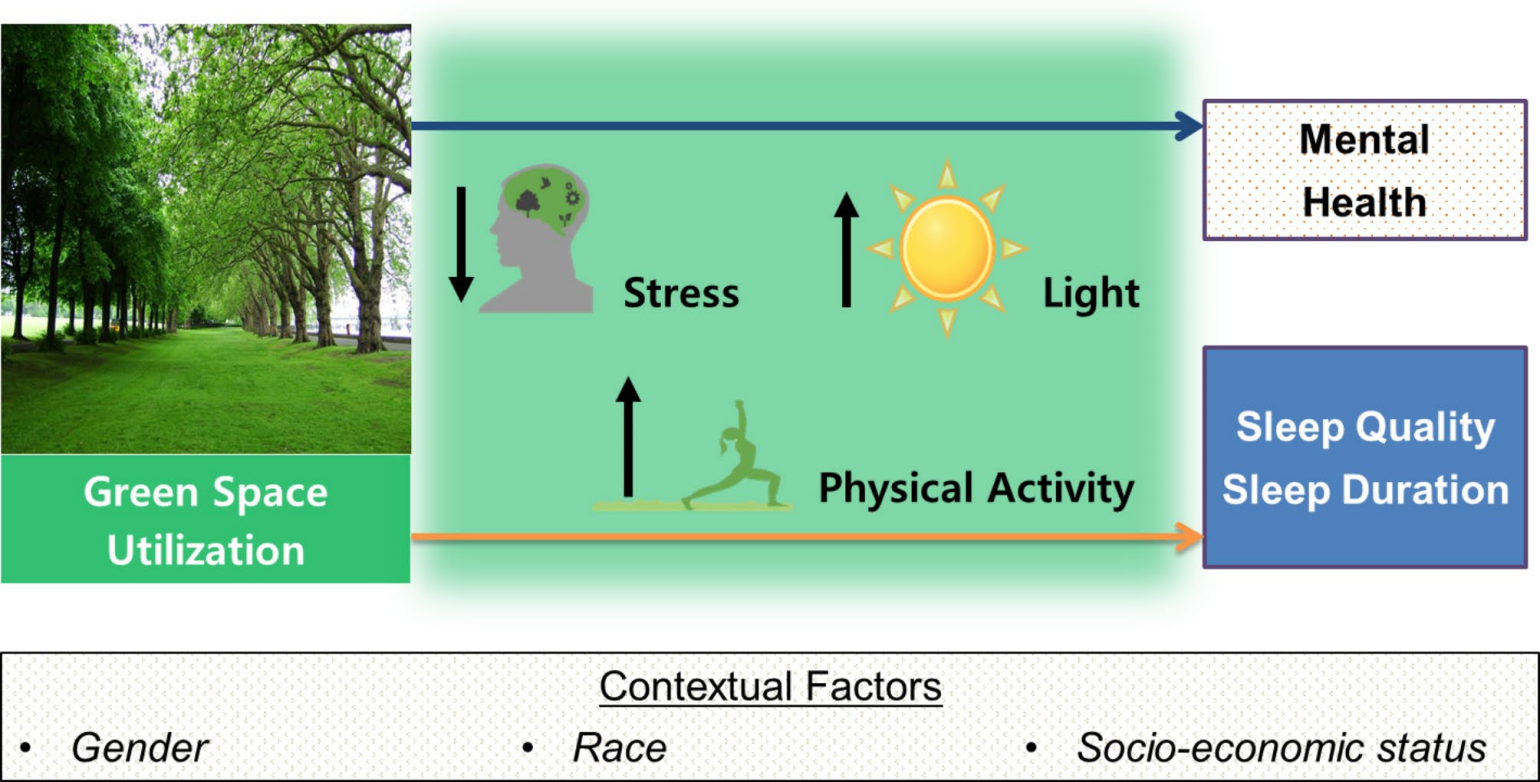
Conceptual Model showing the association between green space exposure, mental health, and sleep

Current theory does not adequately acknowledge the significant role of sleep on children’s mental health, nor has there been extensive research examining the impact of green space on children’s sleep. Here, we expand upon Attention Restoration Theory [51] and Psycho-physiological Stress Reduction Theory [52, 53] which propose that exposure to green space fosters psychological restoration and enhances feelings of relaxation and well-being through exposure to rich, natural stimuli [35]. We theorize that green space influences mental health by reducing stress, increasing exposure to light during the day, and increasing physical activity ultimately leading to better sleep quality, longer sleep duration, and better mental health outcomes (Fig. 1).

#### Green space, mental health, and epigenetics

Green space is understood to have an impact on psychological outcomes, including overall mental well-being [54]. It is noteworthy that epigenetic modifications, such as DNA methylation, partly explain the influence of green space on child neurological development. Particularly relevant to neurodevelopment, early life green space exposure is associated with increased methylation of a cytosine-guanine dinucleotide (CpG) site of the *HTR2A* gene, which is linked to cognitive outcomes such as attention, self-regulation, and neurological processing [55, 56]. Additional studies have linked greenness to methylation patterns of CpG sites with other genes involved in mental health, such as the *CNP* gene. Green space exposure was associated with increased expression of *CNP*, which reduces the risk of schizophrenia and depression. Furthermore, among children, green space exposure was linked with DNA methylation at the *SLC6A3* gene, which is linked to cognition and IQ scores as young as age six [57]. Other notably reported genes associated with greenness exposure, epigenetic changes, and mental health include *PDE4D*,* PLCL1*,* GNG12*, and *SLC6A4*, which are involved in neurotransmitter clearance important for healthy brain development [57]. This study seeks to further explore and contribute additional findings regarding the combined impact of childhood green space exposure on sleep and mental health and related epigenetic associations.

### Aims

The primary objective of this study is to examine how green space, a social determinant of health, contributes to the promotion of mental health, sleep, and wellbeing among elementary school children. The study aims are to (1), to determine how green space utilization is related to sleep (2), to explore mechanisms linking green space to sleep and mental health outcomes and (3) to explore the epigenetic underpinnings of green space on sleep and mental health among children.

## Methods/design

### Sample size calculation

The sample size was determined to ensure sufficient power (> 80%) for the primary and secondary study aims. Power calculations were run using a combination of R and Mplus. Monte Carlo simulations assumed sleep outcomes were correlated at *r* = 0.10, the mechanisms at *r* = 0.20, and the mental health outcomes at *r* = 0.20. Nine correlated (*r* = 0.20) confounders were included and associated with green space engagement, mechanisms (light, stress, physical activity), sleep outcomes, and mental health outcomes at *r* = 0.20. Assuming a Type-I error rate of 0.05, our proposed sample (*N* = 250) will be powered at 0.80 to detect a standardized regression coefficient of 0.17 for the direct effect between green space utilization and sleep behavior and 0.08 for the indirect effect through mechanisms to sleep behaviors, which is akin to an estimate of 0.29 for the relationship between green space utilization and the mechanisms and 0.30 between mechanisms and the sleep outcomes. Power calculations for the secondary aim showed > 80% power given a sample size of *N* = 250 enrolled at baseline. The size of these relationships is smaller than those reported in the literature on green space [58, 59].

### Participants

The target population consists of eligible first, second and third graders, and their primary caregiver, residing in the state of Rhode Island. The inclusion criteria encompass individuals who meet the following conditions: (1) Legal parent or guardian or recognized caregiver (e.g., grandparent) aged 18 years of age or older, (2) Child enrolled in the first, second, or third grade in Rhode Island, (3) Ability to communicate in English or Spanish, and (4) Residence in selected portions of the sampling area to reflect varying levels of green space exposure. The exclusion criteria consist of the following: (1) Disability limiting physical activity, (2) Seizures or other neurological or neuromuscular disorders, (3) Medical condition limiting participation in the study, (4) Not a resident in a sampling area.

### Recruitment procedures

Participants for this study will be recruited through flyer advertisements distributed by their respective schools within Rhode Island, as well as online via social media advertisements posted on Instagram and Facebook. Materials are also translated to Spanish to ensure accessibility for Spanish-speaking individuals. Individuals interested in participating in the study can fill out their information on the flyer and return it to the school for later collection by the lab. Alternatively, individuals can inquire about participation in the study by calling or emailing the lab using the information provided on the social media poster. In either case, upon contact with the lab, individuals will be administered a screener to determine their eligibility for participation in the study based on the previously outlined inclusion and exclusion criteria. Once an individual is found to be eligible to participate in the study, they will be scheduled to meet with the lab for approximately one hour to undergo the informed consent/assent process and begin data collection. Data collection will proceed in the fall (beginning of school until daylight savings) and spring (daylight savings until end of school) to address seasonal changes in light exposure and activity.

### Measures

#### Actigraphy

Participants will wear a multimode actigraph (Actiwatch Spectrum Plus, Philips) on the non-dominant wrist which will record activity and light exposure (photodiode, wavelength range 400–800 nm, peak response 520 nm, measured in Lux) in 15-second epochs. To be included in the data analysis, minimum wear time will be defined as four days during the one-week data collection period.

#### Mental health, family, and parenting measures

Selected scales from the Patient Reported Outcome Measurement Information System (PROMIS^®^) [60–65] will be used to assess child mental health, as reported by parents, at the beginning of the study. These measures are psychometrically sound and are available in Spanish. A variety of children’s mental health domains will be assessed through parent-report subscales, covering areas such as Anxiety, Depressive Symptoms, Cognitive Functioning, Physical Function Mobility, Family Relationships, Psychological Stress, Anger, and Positive Affect among others. In addition to mental health measures, parents will complete surveys regarding their child’s sleep behavior, including the PROMIS© sleep-related impairment and sleep disturbance scale, child sleep habits questionnaire, CCTQ parent proxy chronotype, and the pediatric sleep questionnaire. Parents will also complete the perceptions of green space questionnaire, and an everyday discrimination scale [66], the SPARK survey which will assess physical activity support (e.g., how often an adult engages in physical activity with a child) [67], and a modified version of the youth risk behavior survey (YRBS) to assess screen time. Furthermore, parents will complete a daily activity survey in which they will report family activities and routines such as chores, bedtimes, mealtimes, work and school schedules, etc. Additionally, they will complete daily sleep diaries twice per day, once in the morning and once at night. The morning sleep diary will prompt parents to provide information regarding their child’s previous night of sleep, including perceived sleep duration and sleep onset. The night sleep diary will prompt parents to provide information regarding their child’s activities during the day, including whether they engaged in physical exercise, if they removed the actigraph, and whether they followed a special bedtime routine that night. Survey design and distribution will be completed through the Research Electronic Data Capture (REDCap) tools hosted by Brown University [68, 69].

#### GPS data collection

GPS tracking will be conducted using the Qstarz BT-Q1000XT device, which will record participants’ latitude and longitude every 5 s for 7 days to determine green space exposure. This GPS receiver is highly accurate and equipped with the Wide Area Augmentation System. It is small and lightweight, with a large storage capacity (400,000 waypoints) and a relatively long battery life (42 h). Additionally, it requires minimal attention from participants, as it only needs to be charged overnight. Before the activity-space construction, the GPS and accelerometer data will be processed using the Q-travel program and R. The data will be synchronized within a 2-second differential to build a match that combines activity data from accelerometers with location data from GPS receivers.

#### Saliva and DNA

IL-1β collected using saliva samples will be used as a biomarker of stress. Saliva samples will be collected on the first and last day of the data collection period. Participants will have their DNA collected using a simple and child-friendly method of buccal cell collection.

#### Anthropometrics

Participants’ weight will be measured using a body fat monitor scale from TANITA (model BF-679 W). Height will be measured using a stadiometer from SECA (model 213).

**Fig. 2:**
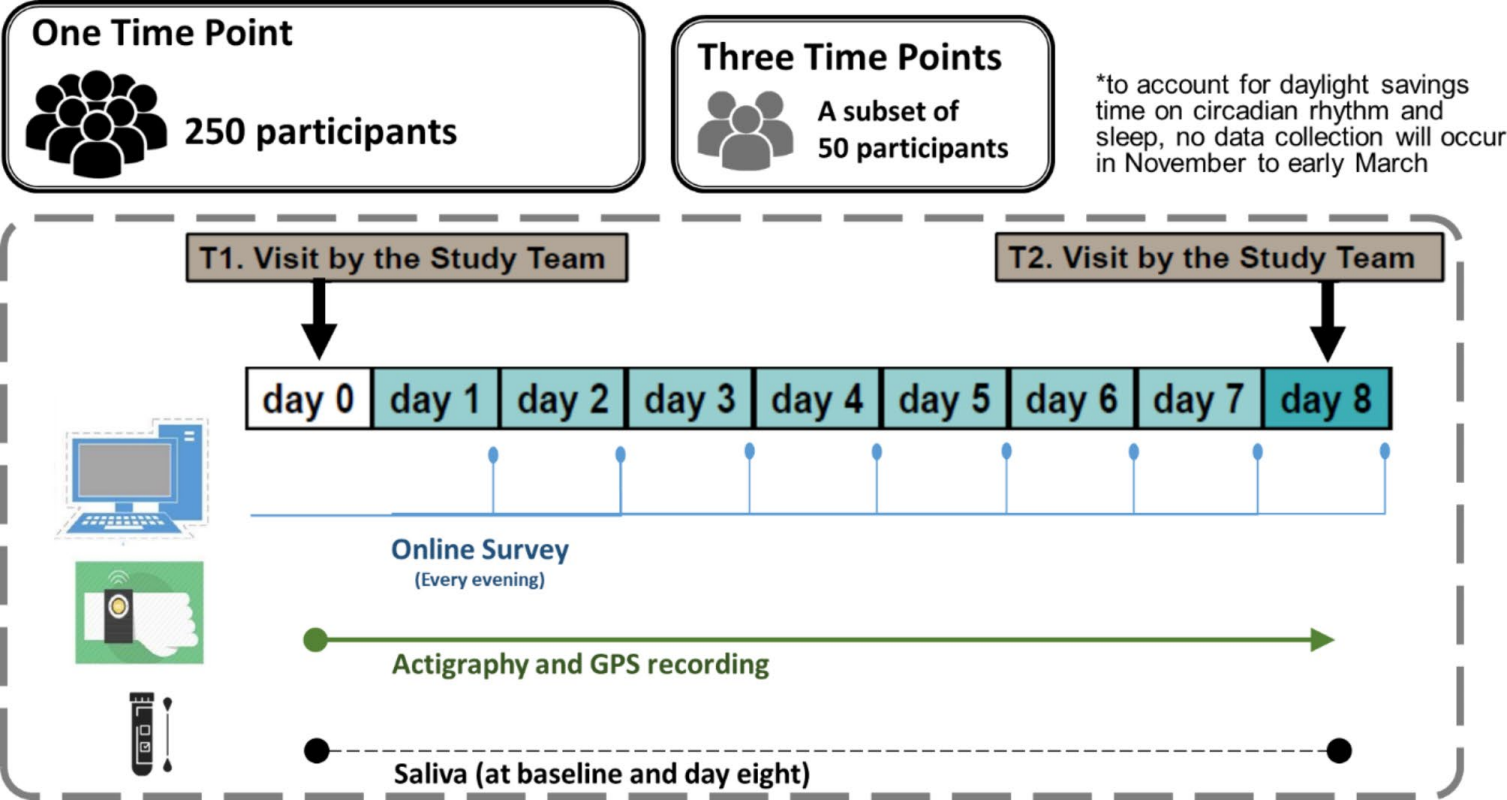
Overview of study procedures and timeline

A summary of data collection procedures can be found in Fig. 2.

### Statistical analysis plan

Descriptive statistics will be used to describe the study sample (means/standard deviations, medians/IQR as appropriate) and graphical methods used to assess the distribution of each of the primary and secondary study outcomes. Missing data patterns will be assessed and methods to address the missingness will be chosen accordingly (including pattern-mixture modeling, multiple imputation, likelihood based approaches to estimation).

### Limitations

To reduce attrition, we hope that remuneration for time and effort will minimize dropouts. Moreover, we plan to address selection bias by examining whether differences exist between the elementary school children who do and do not agree to participate in our study. If differences are found, we will make statistical adjustments to account for bias. We have also focused on sleep behavior, as the use of biological markers to examine the influence of light exposure on the circadian regulation system was not feasible for this study. Notably, sleep behavior is influenced by the circadian system, but does not represent a direct measure of it.

## Discussion

Understanding the impact of green space on children is important given the potential for long-term benefits through supporting or enhancing developmental trajectories related to sleep and mental health (e.g., psychological stress, cognitive function, and academic performance). A better understanding of where children utilize green space as part of their daily routines provides a spatially sophisticated way to identify linkages between inequality and the environment—namely how the conditions in the environment a child interacts with each day shape risks and the degree of access to resources which influence health. Results from our work will assist community leaders in deciding how best to support child development, particularly in areas with limited access to green space. This project is among the first to foster an interdisciplinary collaboration to examine the role epigenetics may play in conferring health benefits of green space on sleep among children. To better understand the mechanisms through which green space confers health benefits on children, we will use environmental and genome-scale data to examine epigenetic modifications associated with green space exposure.

We hope that the unique perspective provided by our study will inform longitudinal study designs to determine whether the influence of green space on sleep is robust, as well as interventions to improve and expand the use and availability of green space among diverse populations of children.

## Data Availability

No datasets were generated or analysed during the current study.

## Declarations

### Clinical trial registration

This is not a clinical trial, as such, there is no TRN.
